# Genotype-Phenotype Analysis and Mutation Spectrum in a Cohort of Chinese Patients With Congenital Nystagmus

**DOI:** 10.3389/fcell.2021.627295

**Published:** 2021-02-19

**Authors:** Xiao-Fang Wang, Hui Chen, Peng-Juan Huang, Zhuo-Kun Feng, Zi-Qi Hua, Xiang Feng, Fang Han, Xiao-Tao Xu, Ren-Juan Shen, Yang Li, Zi-Bing Jin, Huan-Yun Yu

**Affiliations:** ^1^School of Ophthalmology and Optometry, The Eye Hospital, Wenzhou Medical University, Wenzhou, China; ^2^Beijing Ophthalmology and Visual Sciences Key Laboratory, Beijing Institute of Ophthalmology, Beijing Tongren Eye Center, Beijing Tongren Hospital, Capital Medical University, Beijing, China

**Keywords:** congenital nystagmus, *FRMD7*, *GPR143*, mutation, genotype-phenotype

## Abstract

**Purpose:** Congenital nystagmus (CN) is a genetically and clinically heterogeneous ocular disorder that manifests as involuntary, periodic oscillations of the eyes. To date, only *FRMD7* and *GPR143* have been reported to be responsible for causing CN. Here, we aimed to identify the disease-causing mutations and describe the clinical features in the affected members in our study.

**Methods:** All the subjects underwent a detailed ophthalmic examination. Direct sequencing of all coding exons and splice site regions in *FRMD7* and *GPR143* and a mutation assessment were performed in each patient.

**Results:** We found 14 mutations in 14/37 (37.8%) probands, including nine mutations in the *FRMD7* gene and five mutations in the *GPR143* gene, seven of which are novel, including c.284G>A(R95K), c.964C>T(P322S), c.284+10T>G, c.901T>C (Y301H), and c.2014_2023delTCACCCATGG(S672Pfs^*^12) in *FRMD7*, and c.250+1G>C, and c.485G>A (W162^*^) in *GPR143*. The mutation detection rate was 87.5% (7/8) of familial vs. 24.1% (7/29) of sporadic cases. Ten mutations in 24 (41.7%) non-syndromic subjects and 4 mutations in 13(30.8%) syndromic subjects were detected. A total of 77.8% (7/9) of mutations in *FRMD7* were concentrated within the FERM and FA domains, while all mutations in *GPR143* were located in exons 1, 2, 4 and 6. We observed that visual acuity tended to be worse in the *GPR143* group than in the *FRMD7* group, and no obvious difference in other clinical manifestations was found through comparisons in different groups of patients.

**Conclusions:** This study identified 14 mutations (seven novel and seven known) in eight familial and 29 sporadic patients with congenital nystagmus, expanding the mutational spectrum and validating *FRMD7* and *GPR143* as mutation hotspots. These findings also revealed a significant difference in the screening rate between different groups of participants, providing new insights for the strategy of genetic screening and early clinical diagnosis of CN.

## Introduction

Nystagmus is an involuntary, periodic oscillation of unilateral or bilateral eyes. It can be classified into congenital nystagmus (CN) and acquired nystagmus according to the age at onset. The prevalence of nystagmus in the general population has been estimated to be 24/10,000 of the population, and CN is the most common type of all forms of nystagmus (Sarvananthan et al., 2009; Watkins et al., 2012). CN usually appears at birth or in early childhood, and is predominantly characterized by horizontal pendular or jerk nystagmus with various degrees of visual impairment. Abnormal head position (AHP) that is often linked to an eccentric horizontal null position can be observed in patients with CN (Watkins et al., 2012; Brodsky and Dell'Osso, 2014; Papageorgiou et al., 2014; Richards and Wong, 2015). Previous studies have demonstrated that CN may occur as an isolated trait or may be accompanied by other ocular abnormalities such as ocular albinism, strabismus, aniridia, achromatopsia, congenital cataract, Leber congenital amaurosis, retinitis pigmentosa, cone-rod dystrophy and optic nerve hypoplasia (Sarvananthan et al., 2009; Brodsky and Dell'Osso, 2014; Richards and Wong, 2015). At present, although there have been a large number of studies focusing on CN over the past years, the mechanisms of pathogenesis remain unclear. A review in 2015 concluded two main hypotheses to explain this phenotype: dysfunction in the ocular motor control pathways and developmental abnormalities in the anterior visual pathway (Richards and Wong, 2015). Currently, no cure is available for CN, but many treatments, including non-surgical options (prisms, contact lenses, and afferent stimulation) and surgical extraocular muscle surgery, have been reported to improve visual acuity by directly or indirectly reducing but not eliminating nystagmus (Dell'Osso, 2002; Hertle et al., 2010).

Multiple modes of inheritance of CN have been reported in the literature, and X-linked CN is the most common form of hereditary nystagmus with significant clinical and genetic heterogeneity (Forssman, 1971). To date, only *FRMD7* and *GPR143* are considered the major disease-causing genes for CN. However, a recent study found that mutations within the C-terminal region of CASK disrupt the interaction, which is crucial for correct development of oculomotor control between *FRMD7* and CASK, leading to nystagmus (Schnur et al., 1998; Watkins et al., 2013). Mutations were first identified in the *FRMD7* (Xq26-27) gene in both X-linked and sporadic CN cases in 2006 (Tarpey et al., 2006). Since then, more than 90 mutations in the *FRMD7* gene have been reported to date. G-protein coupled receptor 143 (*GPR143*), also known as OA1, was primarily described to cause ocular albinism with nystagmus as a prominent concomitant symptom. Pigmentation loss in the iris and retina is usually not obvious in Asian individuals, therefore, nystagmus may be the main manifestation in patients with *GPR143* mutations (Zhou et al., 2008). Currently, over 100 mutations in *GPR143* have been collected in the HGMD (Human Gene Mutation Database) (http://www.hgmd.cf.ac.uk). The mutation detection rate was found to be 20–57% and 62.5–95% in *FRMD7* and *GPR143*, respectively, in X-linked cases, and much higher than the rates in sporadic cases. The genetic causes of sporadic cases are still poorly understood (Richards and Wong, 2015; Jia et al., 2017a).

In our study, *FRMD7* and *GPR143* mutation analysis and detailed clinical characteristics evaluation were performed in a group of Chinese patients with CN. A total of 9 mutations in the *FRMD7* gene and five mutations in the *GPR143* gene were identified in this study, including 7 novel and 7 previously reported mutations.

## Methods

### Patient Enrolment and Clinical Evaluation

This study was in compliance with the Declaration of Helsinki and was approved by the Institutional Review Board. Informed written consent was obtained from all participants. All participants were enrolled from The Affiliated Eye Hospital of Wenzhou Medical University in this study. The average age was 12 ± 10 years, ranging between 1 year and 46 years old. After a detailed ophthalmic examination including visual acuity, intraocular pressure measurement, a slit-lamp examination, fundus photography, OCT and other specialist review. All the patients were diagnosed with congenital nystagmus by a specialist. Among these participants, 24 were non-syndromic CN cases, 13 were syndromic CN cases (11 cases with strabismus, four cases with ocular albinism, and two patients suffered from both strabismus and ocular albinism).

### DNA Extraction

Dna was extracted from each participant's peripheral blood using a DNA extraction kit (TIANGEN, Beijing, China) following the manufacturer's instructions. Nanodrop 2000 (Thermal Fisher Scientific, Delaware, USA) was used to determine the concentration and purity of the extracted DNA.

### Mutation Screening

The primers of all coding exons and splice site regions in *FRMD7* and *GPR143* were obtained from previous literature (Zhang Q. et al., 2007; Hu et al., 2011). After PCR amplification and direct sequencing of the coding regions and splice site junctions in *FRMD7* and *GPR143*, we analyzed the sequencing of results. The reference genomic sequence versions of *FRMD7* and *GPR143* used were NM_194277.3 and NM_000273.3 from the GenBank database. The potential pathogenicity of the detected mutations in this study was evaluated by the following bioinformatics tools: SIFT (http://sift.jcvi.org/), Polyphen-2 (http://genetics.bwh.harvard.edu/pph2/), Mutation Taster (http://mutationtaster.org/), and PROVEAN (http://provean.jcvi.org/index.php). Allele frequency was assessed by the ExAC database (http://exac.broadinstitute.org/), and 1,000 Genomes Project (ftp://1000genomes.ebi.ac.uk/vol1/ftp). Co-segregation analysis was performed for mutations detected in our study when members in families were available.

### Multiple Sequence Alignment and Molecular Structural Modeling of Missense Mutations

Protein sequences were obtained from the NCBI database (https://www.ncbi.nlm.nih.gov/), and multiple sequence alignments were performed using Clustalx1.83 software. Sequence logos were made with WebLogo3 (http://weblogo.threeplusone.com/). Schematic of protein domain structures were created using DOG 2.0 (Ren et al., 2009). The crystal structures of several wild-type and mutant proteins were predicted by Swiss Model (https://swissmodel.expasy.org/), and the predicted PDB files were visualized by PyMol software (version 2.1.1).

### Statistical Analysis

We used Mann–Whitney U-tests to compare the VA of the two groups. Onset age in two groups was analyzed using a *t*-test. Fisher's exact test was used to evaluate the significance of proportions (strabismus, stereopsis, and AHP) between groups. Non-parametric Kruskal-Wallis tests and Pearson chi-square tests were used to compare multiple groups.

## Results

### Clinical Manifestation

A total of 37 patients with CN (29 male, eight female) were recruited for this study, and the mean age of the participants was 12 ± 10 years. Pedigrees of the 8 (21.6%) families followed an X-linked pattern of inheritance, and the remaining 29 (78.4%) sporadic patients had no positive family history. Different degrees of reduced visual acuity, stereopsis, and AHP were observed among the patients: eleven of them had associated strabismus (29.7%), four of them had associated ocular albinism (10.8%), and two patients had both conditions. A total of 93.8% (30/32) of these patients presented horizontal nystagmus, indicating that horizontal nystagmus was the most common form, which is consistent with previous reports (Zhao et al., 2016).

### Mutation Identified in This Study

**Figure 1:**
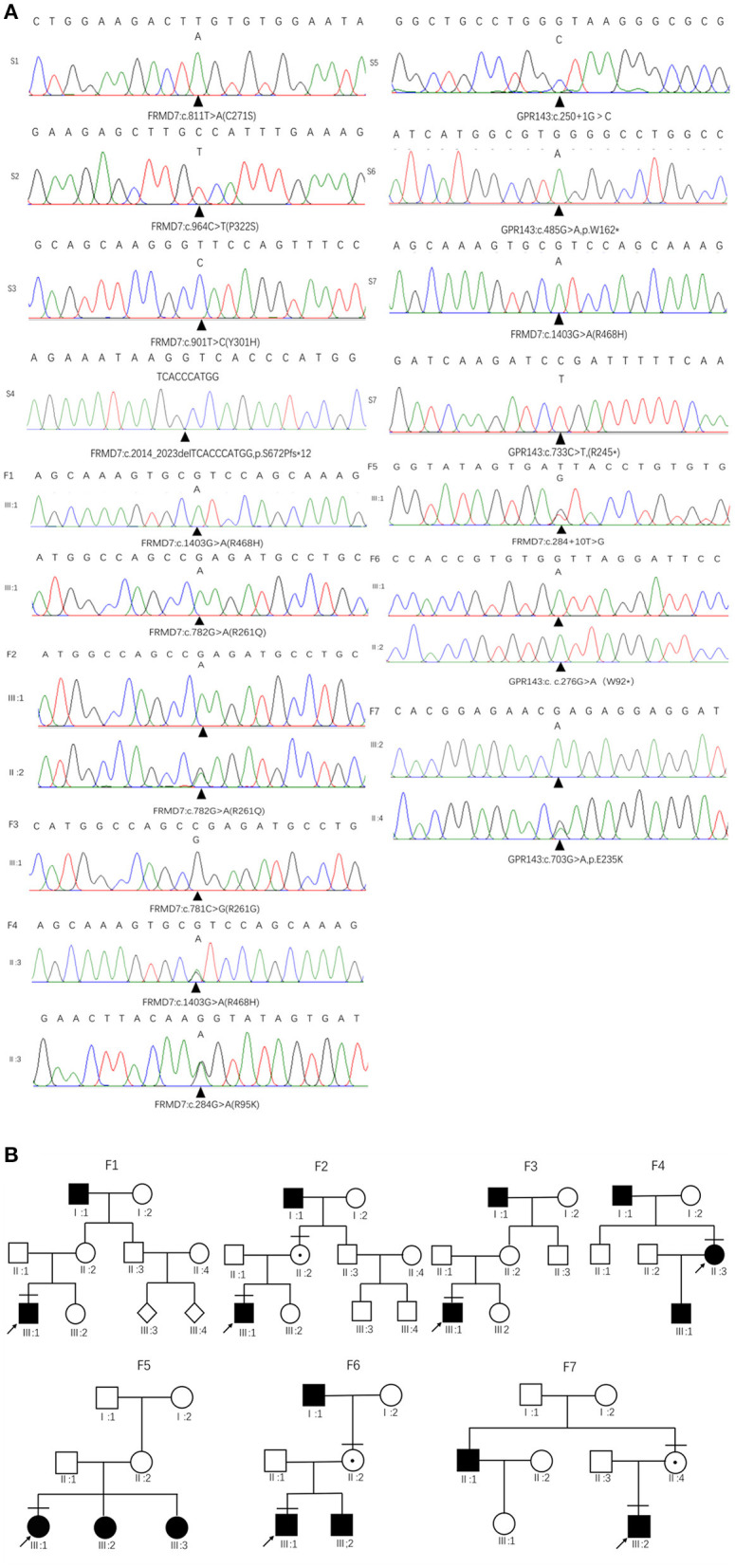
Family pedigrees and Sanger sequencing results. **(A)** The chromatograms of sequencing results: F 1-F 7 represent family 1 to family 7, S 1-S 7 represent sporadic 1 to sporadic 7. **(B)** Pedigrees of *FRMD7* and *GPR143* mutation–positive families: F 1 Family 1; F 2, family 2; F 3, family 3; F 4, family 4; F5, family 5; F 6, family 6; F 7, family 7. Filled symbols indicate affected individuals, unfilled symbols indicate unaffected individuals, and a dotted circle indicates a heterozygous carrier. Bars over the symbols indicate subjects enrolled in this study. Arrows indicate the probands.

**Figure 2:**
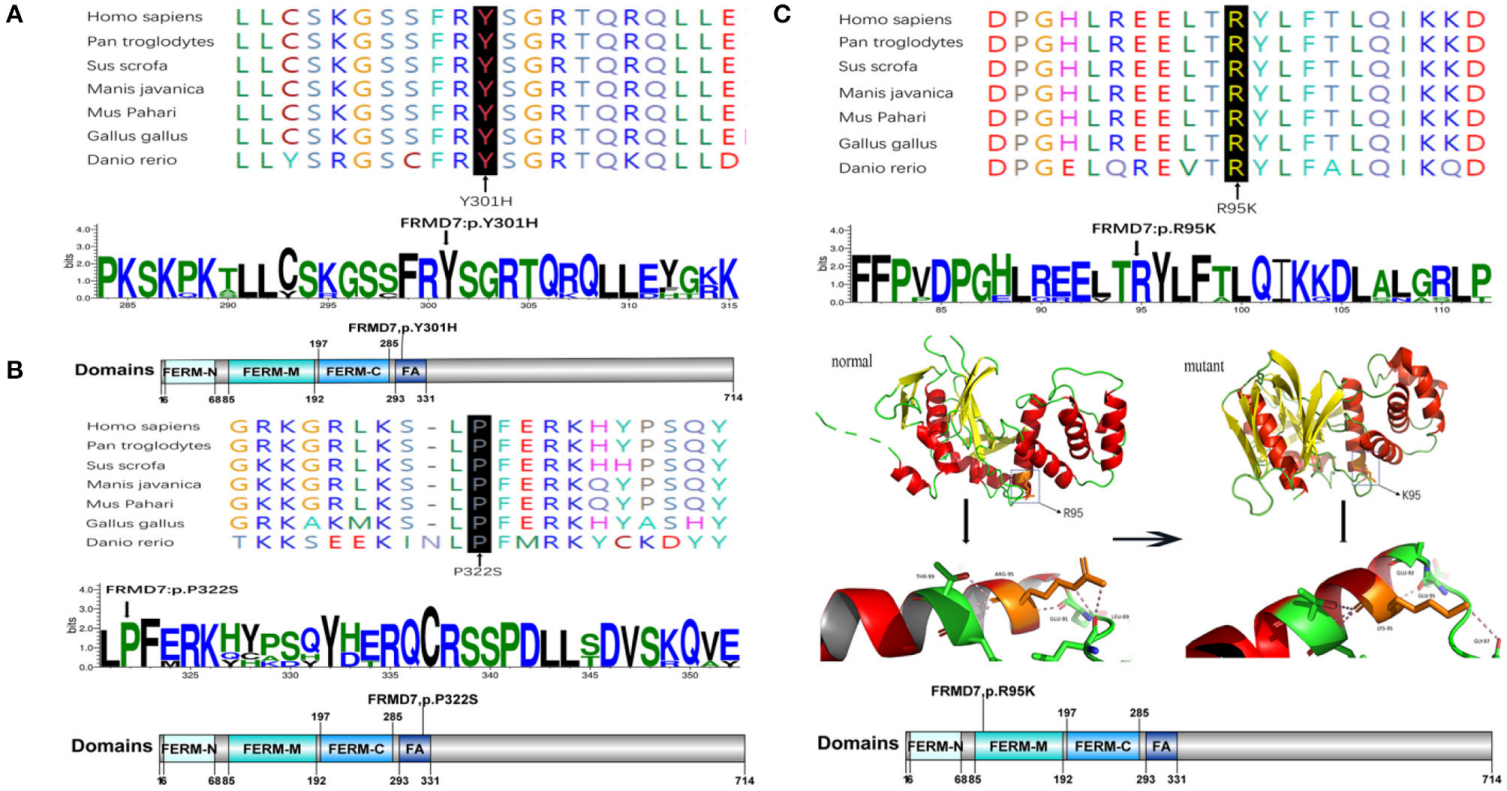
Missense mutations identified in this study. One-dimensional and two-dimensional multiple sequence alignment of the *FRMD7* protein in different species and the predicted three-dimensional structure of the *FRMD7* protein show the high evolutionary conservation of amino acid residues. Black arrows show the location of the novel missense mutations identified in this study in the *FRMD7* protein. **(A)** The alignment of amino acids around residue p.301. **(B)** The alignment of amino acids around residue p.322. **(C)** The alignment of amino acids around residue p.95.The position of the missense mutation c.284G>A around residue p.R95K of *FRMD7* is highlighted in the 3D structure model. Hydrogen bonds between amino acids are shown as pink dashed lines. The normal protein is on the left, and the mutant protein is on the right.

Sanger sequencing of *FRMD7* and *GPR143* in this Chinese cohort revealed 14 different mutations (nine in *GPR143*, five in *FRMD7*) in seven unrelated families and five sporadic cases. Seven mutations are novel: c.284G>A(R95K), c.964C>T(P322S), c.901T>C(Y301H), c.2014_2023delTCACCCATGG(S672Pfs^*^12), and c.284+10T>G in *FRMD7* and c.250+1G>C, and c.485G>A(W162^*^) in *GPR143*. The remaining seven mutations have been reported before. The molecular and clinical results of the 12 participants with identified mutation are shown in Table 1. Mutations identified in our study were assessed for pathogenicity with four different bioinformatics tools, as shown in Table 2. Figure 1 illustrates the pedigrees and the sequencing data. Figure 2 shows the sequence conservation of the *FRMD7* protein of three novel missense mutations and the structural modeling of the novel missense mutation c.284G>A.

**Table 1:** Clinical features of subjects.

**Subject**	**Sex**	**Age(years)**	**Onset age**	**BCVA**	**Nystagmus**	**AHP**	**Accompanying symptoms**	**Family history**	**Mutation gene**
S1	Male	14	1	0.6/0.7	Horizontal jerk	Y	N	N	*FRMD7*
S2	Male	15	2	0.9/0.9	Horizontal jerk	Y	N	N	*FRMD7*
S3	Male	6	1	0.5/0.5	Horizontal jerk	Y	N	N	*FRMD7*
S4	Male	10	At birth	0.6/0.4	Horizontal jerk	Y	N	N	*FRMD7*
S5	Male	17	1	0.3/0.4	Horizontal jerk	Y	Strabismus	Y	*GPR143*
S6	Male	42	2	0.1/0.2	Horizontal jerk	Y	Albinism	N	*GPR143*
S7	Male	22	NA	0.2/0.2	NA	NA	N	N	*GPR143*
F1: III:1	Male	6	1 month	1.0/1.0	Horizontal jerk	Y	N	Y	*FRMD7*
F2: III:1	Male	6	3 months	NA	NA	Y	N	Y	*FRMD7*
F3: III:1	Male	10	1	0.5/0.4	Horizontal jerk	Y	N	Y	*FRMD7*
F4: II:3	Female	33	3	0.8/0.8	NA	N	N	Y	*FRMD7*
F5: III:1	Female	23	3	0.16/0.16	Horizontal jerk	N	Strabismus	Y	*FRMD7*
F6: III:1	Male	6	1	0.1/0.1	NA	N	Albinism	Y	*GPR143*
F7: III:2	Male	7	6 months	0.1/0.1	Horizontal pendular	Y	N	Y	*GPR143*

*Y, present; N, absent; NA, not available*.

**Table 2:** Overview of the mutations found in *FRMD7* and *GPR143* and their assessment.

**Gene**	**Exon/Intron**	**Domain**	**Mutation**	**Protein change**	**Type**	**State**	**Prediction**				**Allele frequency**		**References**
							**Mutation taster**	**PROVEAN**	**SIFT**	**PolyPhen-2**	**1000G**	**EXAC**	
*FRMD7*	9	FERM-C	782G>A	R261Q	Missense	Hemizygous	Disease causing	Deleterious	Damaging	probably damaging	0	0	Li et al., 2008
*FRMD7*	9	FERM-C	781C>G	R261G	Missense	Hemizygous	Disease causing	Deleterious	Damaging	probably damaging	0	0	Zhang B. et al., 2007
*FRMD7*	9	FERM-C	811T>A	C271S	Missense	Hemizygous	Disease causing	Deleterious	Damaging	probably damaging	0	0	Jia et al., 2017b
*FRMD7*	9	FA	**901T>C**	Y301H	Missense	Hemizygous	Disease causing	Deleterious	Damaging	probably damaging	0	0	This study
*FRMD7*	4	FERM-M	**284G>A**	R95K	Missense	Heterozygous	Disease causing	Deleterious	Damaging	probably damaging	0	0	This study
*FRMD7*	Intron 4	FERM-M	**284+10T>G**	-	Splice	Heterozygous	Polymorphism	-	-	-	0	16	This study
*FRMD7*	10	FA	**c.964C>T**	P322S	Missense	Hemizygous	Disease causing	Deleterious	Damaging	probably damaging	0	0	This study
*FRMD7*	12	C-terminal	**2014_2023del TCACCCATGG**	S672Pfs^*^12	Frameshift	Hemizygous	Disease causing	-	-	-	0	0	This study
*FRMD7*	12	C-terminal	c.1403G>A	R468H	Missense	Heterozygous	Polymorphism	Neutral	Damaging	possibly damaging	6	2136	Zhao et al., 2016
*GPR143*	Intron 1	-	**c.250+1G>C**	-	Splice	Hemizygous	Disease causing	-	-	-	0	0	This study
*GPR143*	2	-	c.276G>A	W92^*^	Non-sense	Hemizygous	Disease causing	Deleterious	-	-	0	0	Zou et al., 2017
*GPR143*	4	-	**c.485G>A**	W162^*^	Non-sense	Hemizygous	Disease causing	Deleterious	-	-	0	0	This study
*GPR143*	6	-	c.703G>A	E235K	Missense	Hemizygous	Disease causing	Deleterious	Damaging	probably damaging	0	0	Schnur et al., 1998
*GPR143*	6	-	c.733C>T	R245^*^	Non-sense	Hemizygous	Disease causing	Deleterious	-	-	0	0	Kim et al., 2016

*Novel mutations are highlighted in bold*.

* = X

#### FRMD7 Mutations

We detected a reported missense c.782G>A mutation in exon 9 in family 1 (III1) and family 2 (III1 and II2), which causes the substitution of arginine to glutamine at position 261 (p.R261Q). Li et al. (2008) already identified R261Q in two Chinese families with CN in 2008, and the pathogenicity of this mutation is well-established. In family 2, the unaffected mother of the proband carried c.782G>A, and the results demonstrated the co-segregation of the c.782G>A mutation with CN in family 2. The known missense mutation c.781C>G in exon 9 was found in family 3 (III1), which results in the substitution of an amino acid at a highly conserved position, p.R261G, and has been reported three times in the Chinese population, indicating the high possibility that it is a Chinese-specific variant (Zhang B. et al., 2007; Song et al., 2013; Zhao et al., 2016). The novel missense mutation c.284G>A, leading to a replacement of arginine by lysine at position 95, was detected in a female proband in family 4. A different substitution of amino acid arginine to methionine at 95 has ever been reported by Bai et al. (2017). The substitution occurred in the highly conserved domain FERM-M, and the predicted three-dimensional structure showed a change in the mutant protein. The Arg-95 residue was predicted to locate in the alpha helix of the FERM domain, and the side chain of Arg-95 projected outwards from the alpha helix forming hydrogen bonds with the side chains of Leu-89. In the mutant protein, two hydrogen bonds disappeared between Lys-95 and Leu-89, and Lys-95 side chains formed another one hydrogen bond with the amino acid residues Glu-92 and Gly-87, respectively (Figure 2C). Furthermore, all four software tools predicted the p.R95K mutation to be pathogenic (Table 2). In family 5, the heterozygous donor splice site mutation c.284+10T>G in intron4 was identified in the female proband (III1). This variant was considered to be possibly pathogenic in this family, because the frequency was < 0.01. The other two family members (III2, III3) who were unable to give a peripheral blood sample in this study also showed similar clinical signs of CN and esotropia. In sporadic cases, four mutations, c.811T>A, c.901T>C, c.964C>T, and c.2014_2023delTCACCCATGG, were found in S1, S2, S3, and S4, respectively; the latter three are first reported here. The missense mutation c.811T>A in exon 9 which caused a protein change at p.C271S, has been described before in a previous Chinese study (Jia et al., 2017b). The two novel missense variants resulted in amino acid substitutions at p.Y301H and p.P322S, and both of them occurred in the highly conserved residue of the FERM-adjacent (FA) domain. Multiple sequence alignment suggested that these sites were evolutionarily conserved from Danio to humans (Figures 2A,B). The 10-bp deletion mutation (c.2014_2023delTCACCCATGG) in exon 12 caused a frameshift in the ORF, leading to pre-mature translation termination of the *FRMD7* protein at position 683 (p. S672Pfs^*^12) (Figure 1A). Apart from the mutations above, the c.1403G>A mutation that changed arginine to histidine at position 468 (p.R468H) was identified in three unrelated probands [III1 in family 1, III1 in family 4 and sporadic 2 (S2)] in our study, which was predicted to be non-pathogenic by PROVEAN and Mutation Taster and damaging by SIFT and PolyPhen-2. Based on the fact that its minor allele frequency (MAF)>0.01 and all three probands harbored another disease-causing mutation, in addition to published report (Zhao et al., 2016), the c.1403G>A mutation did not appear to be the main causative mutation in the three probands.

#### GPR143 Mutations

We detected the known non-sense mutation c.276G>A in exon 2 in the proband (III1) and his unaffected mother (II2) in family 6, which introduced a pre-mature stop codon into the ORF and generated a truncated protein. In family 7, III2 and his asymptomatic mother had the missense mutation c.703G>A (from glutamate to lysine), which was first described in a North American family (Schnur et al., 1998). All the pedigrees in which mothers' blood samples were available are consistent with X-linked transmission. In sporadic patients, two new mutations, a splice mutation c.250+1G>C on intron1 and a non-sense mutation c.485G>A truncating the translated protein at position 162, which were not present in the 1,000 Genomes Project or ExAC database and assessed to be pathogenic by prediction tools, were identified in S5 and S6. The known non-sense mutation c.733C>T created an early termination in the *GPR143* protein was present in S7.

### Genotype-Phenotype Correlation Analysis

In our cohort of 37 participants with CN, 9 patients with *FRMD7* mutations and five patients with *GPR143* mutations were identified here. To further investigate the relationship of phenotype-to-genotype, we compared whether there was a statistical difference in terms of clinical characteristics between different groups of patients.

#### Onset Age

We found no significant difference (p>0.05) in onset age was observed between the *FRMD7* group and the *GPR143* group (*t*-test, *P* = 0.2846) or between the *FRMD7* group and the non-*FRMD7* group (*t*-test, *P* = 0.4009 > 0.05). The mean age of onset in our study was later than that in other studies.

#### Visual Acuity

We tested eight of nine patients (accurate visual acuity was not available for one child) in the *FRMD7* group and five patients in the *GPR143* group. The median logMAR visual acuity was −0.22 and −1.00 in these two groups of patients, respectively. The group with *GPR143* mutations had worse vision than in the group with *FRMD7* mutations (Mann–Whitney *U*-test, *P* = 0.0062 < 0.05). However, we did not see an obvious difference in patients' acuity between the *FRMD7* group and non-*FRMD7* group (Mann–Whitney *U*-test, *P* = 0.5279 > 0.05).

#### Strabismus

Only 1 case (11.11%) had esotropia in the *FRMD7* group. By comparison, in the *GPR143* group, one patient (20%) had exotropia, and two patients (40%) had ocular albinism (OA). No observable difference was found in the incidence of strabismus between the two groups (Fisher's exact test, *P* > 0.99). In the non-*FRMD7* group, strabismus was identified in nine of 23 (39.13%) patients (five with esotropia, four with exotropia), and no significant difference was observed between the *FRMD7* group and the non-*FRMD7* group (Fisher's exact test, *P* = 0.21 > 0.05).

#### Stereopsis

Seven of nine subjects were tested for stereopsis in the *FRMD7* group, and one subject (14.29%) with by esotropia demonstrated no stereopsis by the TNO and Titmus tests. In the *GPR143* group, two patients (100%) (test data were not available in the other three patients) with OA and exotropia were found to have no stereopsis in either TNO or Titmus test. In the non-*FRMD7* group, 59.09% (13/22) and 54.55% (12/22) did not have stereopsis by TNO test and Titmus test, respectively, and 61.54% (8/13) and 58.33% (7/12) of which had manifested strabismus in the TNO and Titmus tests, respectively. Fisher's exact test was performed between the *FRMD7* group and non-*FRMD7* group (*p* = 0.08 > 0.05), and statistical comparison between the *GPR143* group and *FRMD7* group was not considered due to the meager data.

#### Anomalous Head Posture

AHP was recorded in 7 (77.78%) patients in the *FRMD7* group compared with 3 (75%) patients in the *GPR143* group (Fisher's exact test, *P* > 0.99). Likewise, a similar proportion of AHP was achieved between the *FRMD7* group (7/9, 77.78%) and the non-*FRMD7* group (13/23, 56.52%) (Fisher's exact test, *p* = 0.42 > 0.05).

In addition to the subjects in our study, we also reviewed all samples as well as mutations in *FRMD7* reported in literature and conducted correlation analysis on patients with CN.

A total of 110 patients were recorded in the literature. We conducted two sets of analysis depending on the mutation locations and types in the patients in terms of VA, onset age, and the proportion of AHP. One-way analysis of variance (ANOVA) was performed and there were no significant differences among the FERM domain group, FA domain group, and other domain group (Kruskal-Wallis test, *p* > 0.05; Pearson chi-square test, *p* > 0.05). Similarly, we did not observe any differences among the missense group, non-sense group, deletion/insertion group, and splice group (Kruskal-Wallis test, *p* > 0.05; Pearson chi-square test, *p* > 0.05).

## Discussion

In this study, a total of fourteen mutations (nine mutations in the *FRMD7* gene and five mutations in the *GPR143* gene) in 37.8% (14/37) of the probands were identified. Seven of them were new mutations.

The mutation detection rate differs significantly between the familial cases (87.5%, 7/8) and the sporadic cases (24.1%, 7/29), and is higher than published studies. These data further support that *FRMD7* and *GPR143* are two major causative genes for CN. Tarpey et al. (2006) first detected mutations in 84.6% (22/26) of the familial cases and 7% (3/42) of the sporadic cases. Zhang Q. et al. (2007) detected mutations in 4 of 14 (28.5%) Chinese families with X-linked nystagmus. Self et al. (2007) identified mutations in 20% (2 of 10) of apparent X-linked families and 3.6% (1/28) singleton cases. A total of 33.3% (7/21) familial and 14.3% (4/28) of sporadic cases had mutations. In 2015, 40% (2/5) of X-linked IN families were found to have mutations, while no mutations were identified in 15 sporadic cases (AlMoallem et al., 2015). The screening rate was 38.89% (7/18) in another Chinese cohort published in 2017 (Jia et al., 2017b). Unexpectedly, 41.7% (10/24) of simple cases had mutations, comparable to 30.8% (4/13) of syndromic cases in this study. This illustrates that mutation screening is equally necessary in both X-linked cases and sporadic cases.

The *FRMD7* gene contains 12 exons and encodes a 714 amino acid protein that is composed of an N-terminal FERM domain, FERM-adjacent domain (FA), and C-terminal domain. The conserved FERM domain comprises three tightly folded clover-leaf structures (F1, F2, F3). Despite several studies, the specific functions of *FRMD7* protein are still uncertain. However, the two closest homologous proteins FARP1 and FARP2 have been found to be involved in the regulation of neural development, indicating that *FRMD7* may play a role in this process. A later study discovered that knockdown of *FRMD7* can cause abnormalities in neurite development, partially confirming the above conjecture, thus, mutations in *FRMD7* can influence neuronal functions, consequently leading to nystagmus (Betts-Henderson et al., 2010).

**Figure 3:**
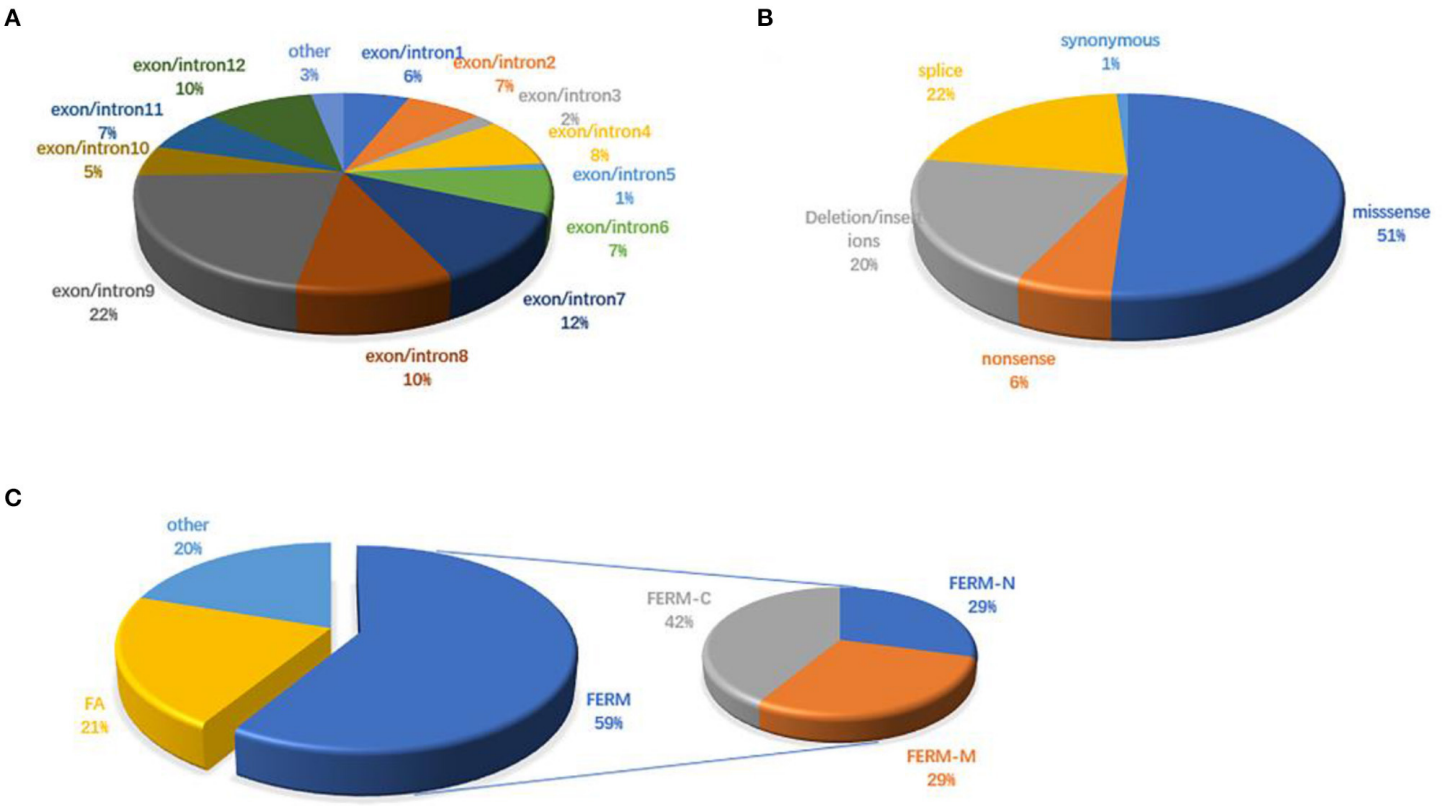
*FRMD7* mutations published in the previous literature. **(A)** Distribution of *FRMD7* mutations identified before on exon/introns. **(B)** Proportions of different types of *FRMD7* mutations. **(C)** Distribution of *FRMD7* mutations identified before on domains in the *FRMD7* protein.

There are 98 mutations reported to date (Supplementary Excel 1 in Supplementary Material). Over half (51.02%) of the mutations are missense and are predicted to change the protein conformation, resulting in affecting the function of *FRMD7*. Non-sense, deletion/insertion, and splice mutations accounted for 6.12, 20.41, and 21.43%, respectively (Figure 3B). A significant number of mutations (80.6%) are primarily concentrated in the highly conserved FERM and FA domains (Figure 3C). In our study, 77.8% (7/9) were missense mutations, and all of them were located in the FERM and FA domains. Notably, the predicted molecular structural modeling of the c.284G>A mutation was similar with the normal protein. Lysine and arginine are both alkaline amino acids with similar molecular properties, but previous study has ever reported mutation of this locus causing nystagmus, and amino acids are well-conserved in the FERM domain, we therefore speculated that this variation of chemical bonds may affect the overall stability and functions of the protein (Bai et al., 2017). Of these 12 exons, exon 9 has been regarded as the mutational hot spot region, containing 21.4% of mutations (Figure 3A). Here, we found a higher proportion, with 4 (44.4%) mutations in exon 9. This indicates that alterations in this region severely affect the function of *FRMD7*. Remarkably, a new 10-bp deletion (c.2014_2023delTCACCCATGG) in exon 12 of C-terminal of *FRMD7* was detected in S4 in our study. As a whole, ten mutations have been identified in exon 12, all of which are predicted to result in severe loss of function of *FRMD7* due to the pre-mature termination of the protein. Only gross structural defects in this region may be responsible for the nystagmus phenotype. Therefore, we speculate that the specific biological function of this region may not be as important as the FERM and FA domains. In addition, we also noticed that c.781C>G and c.782G>A were most frequently reported in the Chinese population, and have not been found in other populations to date. c.782G>A were observed twice in 2 male probands of two unrelated families in our patients. The above findings to some extent suggest that these mutations are unique to Chinese people.

The *GPR143* gene consists of 9 exons and encodes the G protein-coupled receptor 143 that is present on the membrane of melanosomes in pigment cells. Recent research has proven that *GPR143* expression can be detected during the earliest stage of melanosome formation in the RPE. Mutations in *GPR143* may cause isolated albinism in the eye or a series of other abnormalities, such as reduced visual acuity, nystagmus, and strabismus (Surace et al., 2000). The underlying pathogenic mechanisms of ocular abnormities caused by the GPR143 mutation have not been fully studied. It is well-known that melanin synthesis is disrupted in albinism, when this pathogenic condition occurs in the RPE, leading to severe defects in development and maintenance of vision eyes, including foveal hypoplasia, decreased numbers of photoreceptors and ganglion cells and misrouting of the optic tracts at the chiasm. However, mutations in GPR143, one of the genes associated with albinism, result in the impairment of visual pathway, but with intact melanin synthesis machinery (Schiaffino, 2010). This suggests that it is not the melanin synthesis and accumulation but the GPR143 signaling activity responsible for the developmental defects in retina. Researchers proposed that GPR143 signaling was the downstream of pigmentation, and all forms of albinism were dependent upon GPR143 signaling pathway to affect retinal development. Later studies found GPR143 signaling in RPE regulated the secretion of pigment epithelium-derived factor (PEDF) and vascular endothelial growth factor (VEGF) and exosome release. All of these signaling activities were involved in the protection from retinal diseases. However, it remains unclear that how the exosome release influences the retina (Locke et al., 2014; McKay, 2019; Figueroa and McKay, 2020). Mutations in GPR143 contributing to the aberrant protein function might disrupt the GPR143 signaling pathway leading to the developmental visual disorder. It was concluded that mutations in *GPR143* tend to cluster in exons 1-7; similarly, in this paper, all of these mutations in *GPR143* were found in exons 1, 2, 4, and 6. These results suggested that exons 1-7 are the most frequently mutated regions in the *GPR143* gene (Schnur et al., 1998; Fang et al., 2008).

However, several studies have reported that Chinese patients with *GPR143* mutations manifested CN as the most dominant, consistent phenotype. Researchers have proposed that the typical symptoms of OA caused by *GPR143* mutations are seldom observed in Asian populations, mainly because they have dark irises (Table 3) (Preising et al., 2001; Liu et al., 2007; Zhou et al., 2008; Peng et al., 2009; Xiao and Zhang, 2009; Hu et al., 2011; Gao et al., 2019). Three of five mutations (c.703G>A, c.733C>T, c.250+1G>C) in *GPR143* were detected in three cases without ocular albinism in our study. Unlike the study here, c.703G>A was reported to cause typical ocular albinism, while patients with the c.733C>T mutation exhibited foveal hypoplasia in previous studies (Schnur et al., 1998; Kim et al., 2016). In addition, Janecke et al. (2012) reported a patient misdiagnosed with CN first the time who was examined to have slight hypopigmentation in the fundus and was finally identified as ocular albinism through genetic screening. Herein, we summarized all the mutations in GPR143 identified in Chinses populations and phenotypes of the probands. It is believed that nystagmus, foveal hypoplasia, and hypopigmentation in the fundus are all associated with *GPR143* mutations (Table 3). We did not perform OCT to re-evaluate the three subjects more meticulously in our study due to the difficulty in children's examinations. Early and precise diagnosis is crucial to individual management and correct therapy. Several studies have shown that ultrahigh resolution OCT could detect minor differences in structural changes in the retina between albinism- and *FRMD7-* associated nystagmus (Thomas et al., 2014). Thus, comprehensive fundus examinations (OCT, fundus photograph) and molecular analysis are all required to distinguish between CN caused by *FRMD7* mutations and atypical OA caused by *GPR143* mutations more accurately. Targeted screening will be helpful to substantially increase the detection rate.

**Table 3:** Mutations identified in *GPR143* and the clinical characteristics of the probands in the Chinese population.

**Mutation**	**Sex**	**Age(years)**	**BCVA**	**CN**	**Iris hypopigmentation**	**Fundus hypopigmentation**	**Macular hypoplasia**	**References**
c.360+5G>T	Male	23	0.25/0.25	Y	Y	Y	Y	Gao et al., 2019
g.4572_5239del668bp	Male	5	0.2/0.2	Y	Y	Y	Y	Jiang et al., 2019
c.208_218del	Male	10	0.2/0.3	Y	Mild	N	Y	Jiang et al., 2019
g.4709_5711del1010bp	Male	52	0.1/0.1	Y	Mild	Y	Y	Jiang et al., 2019
c.659-2A>C	Male	10	0.1/0.1	Y	Mild	N	Y	Jiang et al., 2019
c.251G>A	Male	9	0.2/0.3	Y	Mild	N	Y	Jiang et al., 2019
c.733C>T	Male	7	0.1/0.1	Y	Mild	N	Y	Jiang et al., 2019
c.333G>A	Male	6 months	LP/LP	Y	Mild	Y	NA	Jia et al., 2017a
c.353G>A	Male	7	0.2/0.2	Y	N	Y	NA	Jia et al., 2017a
c.658+2T>G	Male	8 months	LP/LP	Y	Mild	Y	NA	Jia et al., 2017a
c.215_216 ins CGCTGC	Male	2 months	NA	Y	Mild	Y	NA	Jia et al., 2017a
c.17T>C	Male	10	0.3/0.3	Y	Mild	Y	NA	Jia et al., 2017a
c.333_360+14del42insCTT	Male	4	NA	Y	N	Y	Y	Zou et al., 2017
c.276G>A	Male	3	LP/LP	Y	N	Y	Y	Zou et al., 2017
c.793C>T	Male	24	0.15/0.15	Y	Y	Y	Y	Zou et al., 2017
exon 3_9del	Male	NA	NA	Y	N	N	N	Bu et al., 2016
c.494C>A	Male	4 months	NA	Y	N	Y	Y	Pan et al., 2016
c.333G>A	Female	36	0.1/0.1	Y	N	Y	Y	Han et al., 2015
c.360+1G>C	Male	7	0.1/0.1	Y	N	Y	Y	Han et al., 2015
c.659-1G>A	Male	10	0.3/0.2	Y	N	Y	Y	Han et al., 2015
c.43_50dupGACGCAGC	Male	8	0.3/0.3	Y	N	Y	Y	Han et al., 2015
c.703G> A	Male	29	0.3/0.4	Y	N	Y	Y	Han et al., 2015
g.24422G>C	Male	34	0.3/0.5	Y	Mild	Y	Y	Cai et al., 2013
c.943G>T	Male	6 months	NA	Y	Y	Y	Y	Wang et al., 2009
c.266C>T	Male	18	0.3/0.4	Y	N	N	N	Liu et al., 2007
c.807T>A	Male	42	0.2/0.2	Y	Mild	N	Y	Janecke et al., 2012
exon 1_2	Male	8	0.2/0.2	Y	N	Mild	Y	Xiao and Zhang, 2009
c.658+1G>T	Male	7	0.1/0.2	Y	Mild	Y	Y	Hu et al., 2011
c.849delT	Male	8	0.2/0.2	Y	N	N	N	Fang et al., 2008
c.238_240delCTC	Male	4	0.1/0.1	Y	NA	NA	NA	Fang et al., 2008
c.658+1G>A	Male	4	NA	Y	Mild	N	N	Fang et al., 2008
c.353G>A	Male	7	0.2/0.2	Y	Mild	N	Y	Fang et al., 2008
g.1103_7266del6164bp	Male	12	0.2/0.2	Y	N	N	N	Fang et al., 2008
g.25985_26546del562bp	Male	4	ND	Y	Mild	N	Y	Fang et al., 2008

*Y, present; N, absent; NA, not available*.

We did witness a considerable difference in visual acuity between the *FRMD7*-group and the *GPR143*-group. This result was in keeping with previous observational studies in which patients with *GPR143* mutations suffered more severe damage to vision, which may be explained by sensory defects in albinism-associated mechanisms, although this was not determined till now. GPR143 signaling might be essential for the process of RPE pigmentation protecting neurosensory retina. Although no typical albinism symptoms were observed, GPR143 mutation screening should be required for patients with poor visual function. In contrast to the earlier findings in 2011, comparisons of other clinical characteristics between the two groups are similar in our groups (Kumar et al., 2011).

Thomas et al. has described a lower proportion of AHP in the *FRMD7* group than the non-*FRMD7* group, which was not observed in our study. In addition, the remaining features of strabismus and stereopsis in the different groups here did not exhibit significant differences, which is compatible with previous studies (Thomas et al., 2008; Kumar et al., 2011). Noticeably, 100% of patients in *FRMD7* group and 61.54% (8/13) by TNO test and 58.33% (7/12) by Titmus test in the non-*FRMD7* group manifested strabismus in the group in which no stereopsis was recorded. This implies that strabismus has a strong adverse effect on binocular visual function. Excluding those with strabismus could be beneficial to improve the reliability of stereopsis phenotype analysis in different groups.

Unexpectedly, subjects with different mutation types exhibited no clear phenotypic-mutant link. In our study, we analyzed the clinical features of all the subjects reported before, but we did not conclude any differences among the non-sense, missense, deletion/insertion, and splice mutation groups. It seems that mutation types don not response to the severity of phenotype, and research by Thomas et al. (2008) also supported this finding. Other factors including the environment, modifier genes, and other unknown factors contributing to modulating the disease, may explain the phenotypic diversity.

In this research, we identified 14 mutations in two different genes, *FRMD7* and *GPR143*, and seven of them are novel. However, we cannot exclude the possibility that mutations are present in non-coding regions of *FRMD7* and *GPR143*. The combination of whole-genome and whole-exon sequencing is needed to identify novel candidate genes or variants in our unsolved cases.

However, several limitations in this study need to be acknowledged. First, it was difficult to obtain peripheral blood from some family members, and family segregation analysis was performed in only three families. Second, we did not re-evaluate patients without perfect clinical information, and the sample size may not be large enough, which might affect the credibility of the genotype–phenotype associations. Finally, the pathogenicity of new mutations was predicted by bioinformatic tools, and further experiments on cells and animals were not been performed in this study given the limited time and cost.

In conclusion, despite the limitations of this study, our research certainly provides another large-scale cohort with CN since the first four cohorts (Tarpey et al., 2006; Thomas et al., 2008; AlMoallem et al., 2015; Choi et al., 2018). For the first the time, we compared the phenotypes between groups with *FRMD7* and *GPR143* mutations and reviewed all the patients reported to explore the relationship of mutations and clinical characteristics. Here, 14 mutations were detected in 37.8% (14/37) of the probands (seven familiar cases, seven sporadic cases), seven of which are novel, demonstrating that mutations in *FRMD7* and *GPR143* appear to be two major causative factors in both X-linked and sporadic cases. Worse visual acuity was observed in patients with *GPR143* mutations than in patients with *FRMD7* mutations. These results broaden the mutation spectrum of *FRMD7* and *GPR143*, and add to our knowledge on Chinese patients with congenital nystagmus, providing new insights for the strategy of precise diagnosis and genetic counseling of CN.

## Data Availability Statement

The raw data supporting the conclusions of this article will be made available by the authors, without undue reservation.

## Ethics Statement

The studies involving human participants were reviewed and approved by Ethics Committee of Eye Hospital of Wenzhou Medical University. Written informed consent to participate in this study was provided by the participants' legal guardian/next of kin.

## Author Contributions

Z-BJ conceived, supervised the study, and provided funding supports. H-YY and HC evaluated clinical characteristics for the enrolled patients. X-FW, P-JH, Z-KF, Z-QH, XF, X-TX, FH, and R-JS carried out the experiments. X-FW performed the data analysis and wrote the manuscript. Z-BJ and YL revised the manuscript. All authors contributed to the article and approved the submitted version.

## Conflict of Interest

The authors declare that the research was conducted in the absence of any commercial or financial relationships that could be construed as a potential conflict of interest.
